# Genetic association of deleted in colorectal carcinoma variants with breast cancer risk: A case-control study

**DOI:** 10.18632/oncotarget.9024

**Published:** 2016-04-26

**Authors:** Xinghan Liu, Xijing Wang, Sidney W. Fu, Meng Wang, Huafeng Kang, Haitao Guan, Shuqun Zhang, Xiaobin Ma, Shuai Lin, Kang Liu, Yanjing Feng, Cong Dai, Zhijun Dai

**Affiliations:** ^1^ Department of Oncology, Second Affiliated Hospital of Xi'an Jiaotong University, Xi'an 710004, China; ^2^ Division of Genomic Medicine/Department of Medicine, The George Washington University School of Medicine and Health Sciences, Washington, DC 20037, USA

**Keywords:** DCC, breast cancer, gene variant, susceptibility, case-control

## Abstract

Deleted in colorectal carcinoma (*DCC*), a netrin-1 dependence receptor, is correlated with cell progression, migration, and adhesion. Evidence indicated that *DCC* was frequently down-regulated in many cancers. However, the association of *DCC* with breast cancer remains uncertain. We conducted a case-control study to investigate the impact of three *DCC* gene variants (rs2229080, rs7504990, and rs4078288) on breast cancer susceptibility in Chinese women. This study included 560 breast cancer patients and 583 age-matched healthy controls from Northwest China. The three gene variants were genotyped via Sequenom MassARRAY. Odds ratios (ORs) and 95% confidence intervals (CIs) were utilized to evaluate the associations. We found that individuals with the rs2229080 C/G, C/C, and C/G-CC genotypes had a higher breast cancer risk, and the minor allele C was associated with increased breast cancer risk in an allele model. We observed a significantly decreased breast cancer risk with the rs7504990 C/T, T/T, and C/T-T/T genotypes, and the minor allele T was protective against breast cancer in an allele model. In addition, rs2229080 was associated with the axillary lymph node (LN) metastasis status. An age-stratified analysis revealed an association between rs2229080 and reduced breast cancer risk among older patients (≥ 49 years). Furthermore, the haplotype analysis showed that the C_rs2229080_C_rs7504990_A_rs4078288_ haplotype was associated with a decreased breast cancer risk. However, the results indicated a lack of association between rs4078288 and breast cancer risk. These findings affirmed that rs2229080 and rs7504990 polymorphisms in *DCC* might be related with breast cancer susceptibility in Chinese women.

## INTRODUCTION

Breast cancer (BC) is the most common type of tumors in women worldwide, with 1.8 million incident cases and 464,000 death cases in 2013 [1]. Approximately 5% to 10% of BCs are metastatic at diagnosis, and of these, approximately one-fifth of the affected patients will survive for 5 years [2]. A previous study estimated that the expression of axon guidance molecules (AGMs) was dysregulated during BC tumorigenesis and tumor progression, suggesting that AGMs might act as tumor suppressors and oncogene activators [3].

In the nervous system, axon development is guided by diffusible chemoattractants produced by axonal target cells [4]. Netrins, a family of extracellular proteins, are considered critical axon guidance cues for the positioning of axonal growth during neural circuit formation and various biological processes, including tumorigenesis, adhesion, and angiogenesis [5]. Netrin-1 and netrin-4 are the most frequently studied members of the netrin family [6]. Netrin-1 expression is elevated in human renal clear cell carcinomas relative to normal tissues [7]. Netrin-1 overexpression is considered a poor prognostic factor in ovarian malignancies [8]. A complete loss of netrin-1 causes embryonic death and severe axon guidance defects in mice [9]. Netrin-1 receptors such as *DCC* and SAX-3 (Robo) can function individually or in combination with other guidance receptors to control axon growth [10]. Evidence shows that *DCC* is widely expressed by neurons and is enriched at synapses to promote synaptogenesis between mammalian cortical neurons [11]. In addition, *DCC* can induce apoptosis in the absence of its ligand netrin-1 [12].

Recently researchers are mostly focused on the association between rs2229080, rs7504990 and rs4078288 polymorphisms in *DCC* and many cancers. Rs2229080 is located on human chromosome 18, with a C/G single-nucleotide variation, which may alter the *DCC* expression. Rs7504990 and rs4078288 are located in the intron region and may not directly affect protein function. Rai et al. [13] did not confirm rs4078288 and rs7504990 polymorphisms as the risk factors for gallbladder cancer, different with the results of GWAS. However, to our knowledge, there were no studies involved the association between the three *DCC* polymorphisms and BC susceptibility. In this case-control study, we aimed to examine the association of these three *DCC* gene polymorphisms (rs2229080, rs7504990, and rs4078288) with BC risk in a Northwest Chinese population.

## RESULTS

### Characteristics of the patients and controls

The characteristics of the 560 BC cases and 583 controls are presented in Table 1. The mean ages of patients with BC and healthy controls were 49.09 ± 11.02 and 48.80 ± 8.28 years, respectively. No significant differences were noted between BC cases and controls in terms of the age distribution (*P* = 0.612) and menopausal status (*P* = 0.716). However, the body mass index of BC patients significantly differed from health controls (*P* = 0.038). Therefore, statistical analysis based on case-control comparisons was adjusted for BMI.

**Table 1:** Distributions of select variables in breast cancer cases and cancer-free controls

Characteristics		Cases	Control	*P* value*
**Number**		560	583	
**Age (mean ± SD)**		49.09 ± 11.02	48.80 ± 8.28	0.612
Menopausal status				
**Premenopausal**		264	281	
**Postmenopausal**		296	302	0.716
Body mass index (kg/m^2^)				
**(mean ± SD)**		22.52 ± 2.84	22.95 ± 3.21	**0.038**
**Tumor size**	< 2 cm	188		
	≥ 2 cm	372		
**LN metastasis**	Negative	236		
	Positive	324		
**ER**	Negative	247		
	Positive	313		
**PR**	Negative	255		
	Positive	305		
**Her-2**	Negative	389		
	Positive	171		
**Ki67**	< 14%	195		
	≥ 14%	365		

* *T*-test or two-sided χ^2^-test.

LN: Axillary lymph node; ER: Estrogen receptor; PR: Progesterone receptor; HER-2: human epidermal growth factor receptor 2.

### Association between *DCC* gene variants and BC risk

**Figure 1:**
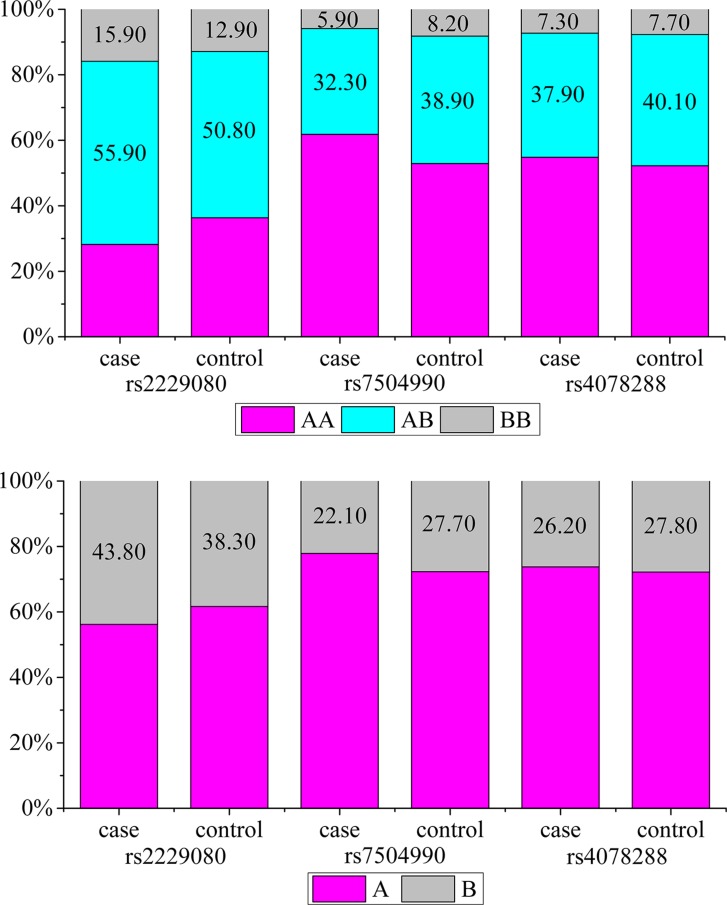
(**A**) Genotype frequencies of the *DCC* polymorphisms among the cases and controls. (**B**) Allele frequencies of the *DCC* polymorphisms among the cases and controls. (A) the major allele; (B) the minor allele.

All three SNPs of DCC gene were successfully genotyped in 560 patients and 583 controls. The genotypes and allele frequencies of the *DCC* rs2229080, rs7504990, and rs4078288 variants are shown in Table 2 and Figure 1. The genotype distributions of the three SNPs in healthy controls exhibited Hardy–Weinberg Equilibrium (HWE) (*P* = 0.07, 0.50, and 0.99 for rs2229080, rs7504990, and rs4078288, respectively), indicating that community genetic inheritance was balanced in the control samples and that these samples could represent the general population.

**Table 2:** Genotype and allele frequencies of the *DCC* polymorphisms among the cases and controls and the associations with breast cancer risk

Model	Genotype	Case (560)	Control (583)	OR (95% CI)^†^	*P*-value*
**rs2229080 HWE = 0.07**					
**Codominant**	G/G	158 (28.2%)	212 (36.3%)	1.00 (reference)	
	C/G	313 (55.9%)	296 (50.8%)	1.42 (1.09–1.84)	**0.008**
	CC	89 (15.9%)	75 (12.9%)	1.59 (1.10–2.31)	**0.013**
**Dominant**	GG	158 (28.2%)	212 (36.3%)	1.00 (reference)	
	**C/G-C/C**	402 (71.8%)	371 (63.7%)	1.45 (1.13–1.87)	**0.003**
**Recessive**	G/G-C/G	471 (84.1%)	508 (87.1%)	1.00 (reference)	
	**C/C**	89 (15.9%)	75 (12.9%)	1.28 (0.92–1.78)	0.144
**Allele**	G	629 (56.2%)	720 (61.7%)	1.00 (reference)	
	C	491 (43.8%)	446 (38.3%)	1.26 (1.07–1.49)	**0.007**
**rs7504990 HWE = 0.50**					
**Codominant**	C/C	346 (61.8%)	308 (52.8%)	1.00 (reference)	
	C/T	181 (32.3%)	227 (38.9%)	0.71 (0.55–0.91)	**0.007**
	T/T	33 (5.9%)	48 (8.2%)	0.61 (0.38–0.98)	**0.039**
**Dominant**	C/C	346 (%)	308 (52.8%)	1.00 (reference)	
	C/T-T/T	214 (38.2%)	275 (47.2%)	0.69 (0.55–0.88)	**0.002**
**Recessive**	C/C-C/T	527 (94.1%)	535 (91.8%)	1.00 (reference)	
	T/T	33 (5.9%)	48 (8.2%)	0.70 (0.44–1.11)	0.123
**Allele**	C	873 (77.9%)	843 (72.3%)	1.00 (reference)	
	T	247 (22.1%)	323 (27.7%)	0.74 (0.61–0.89)	**0.002**
**rs4078288 HWE = 0.99**					
**Codominant**	A/A	307 (54.8%)	304 (52.1%)	1.00 (reference)	
	G/A	212 (37.9%)	234 (40.1%)	0.90 (0.70–1.15)	0.384
	G/G	41 (7.3%)	45 (7.7%)	0.90 (0.57–1.42)	0.655
**Dominant**	A/A	307 (54.8%)	304 (52.1%)	1.00 (reference)	
	G/A-G/G	253 (45.2%)	279 (47.9%)	0.90 (0.71–1.13)	0.364
**Recessive**	A/A-G/A	519 (92.7%)	538 (92.3%)	1.00 (reference)	
	G/G	41 (7.3%)	45 (7.7%)	0.94 (0.61–1.47)	0.799
**Allele**	A	826 (73.8%)	842 (72.2%)	1.00 (reference)	
	G	294 (26.2%)	324 (27.8%)	0.93 (0.77–1.11)	0.408

* Two-sided χ^2^ test for the distributions of genotype and allele frequencies.

† Adjusted for age and body mass index.

Among all selected *DCC* polymorphisms, two were found to be associated with the risk of BC. For the rs2229080 polymorphism, we found that C/G, C/C, and C/G-CC genotype carriers had a significantly increased risk of developing BC (OR > 1, *P* < 0.05); the same trend was observed for patients with the C allele (*P* = 0.007). For rs7504990, patients with BC were less likely than controls to carry the C/T, T/T, and C/T-T/T genotypes (32.3% vs. 38.9%, 5.9% vs. 8.2%, and 38.2% vs. 47.2%, respectively), and the minor allele T conferred a reduced risk to BC in an allele model (OR = 0.74, 95% CI = 0.61–0.89, *P* = 0.002). However, the patients and controls did not differ significantly with respect to the frequency of any rs4704853 genotypes (*P* > 0.05). We also obtained the statistical power of 0.86 and 0.83 for the two significant polymorphisms identified, rs2229080 and rs7504990, respectively. This showed that our sample size of 1143 was adequate and the study was sufficiently able to detect the true association of these two polymorphisms with BC.

### Association between *DCC* gene variants and clinical parameters of patients with BC

To investigate whether *DCC* variants were associated with the clinical features of patients with BC, we further analyzed the distributions of *DCC* variants with respect to a series of clinicopathological parameters including tumor size; axillary lymph node (LN) metastasis; and estrogen receptor (ER), progesterone receptor (PR), human epidermal growth factor receptor 2 (HER-2), and Ki67 index statuses. As shown in Table 3, rs2229080 was associated with LN metastais in patients with BC (*P* = 0.01). However, no significant correlations were detected between the rs7504990 or rs4704853 polymorphisms and clinical features of patients with BC (data not shown).

**Table 3:** The associations between the *DCC* polymorphisms and clinical characteristics of breast cancer patients

Variables	rs2229080				rs7504990			
	G/G (%)	C/G+C/C(%)	P *	OR (95%CI)	CC (%)	C/T+T/T (%)	P *	OR (95%CI)
**Tumor size**								
**< 2 cm**	54 (28.7%)	134 (71.3%)		1.00 (reference)	127 (67.6%)	61 (32.4%)		1.00 (reference)
**≥ 2 cm**	104 (28.0%)	268 (72.0%)	0.85	1.04 (0.70–1.53)	219 (58.9%)	153 (41.1%)	0.05	1.46 (1.01–2.10)
**LN metastasis**								
**Negative**	81 (37.3%)	155 (62.7%)		1.00 (reference)	143 (60.1%)	93 (39.4%)		1.00 (reference)
**Positive**	77 (23.8%)	247 (76.2%)	**0.01**	1.68 (1.16–2.43)	203 (62.7%)	121 (37.3%)	0.62	0.92 (0.65–1.29)
**ER**								
**Negative**	61 (24.7%)	186 (75.3%)		1.00 (reference)	148 (60.0%)	99 (40.0%)		1.00 (reference)
**Positive**	97 (31.0%)	216 (69.0%)	0.10	0.73 (0.50–1.06)	198 (63.3%)	115 (36.7%)	0.42	0.87 (0.62–1.22)
**PR**								
**Negative**	69 (27.1%)	186 (72.9%)		1.00 (reference)	155 (60.8%)	100 (39.2%)		1.00 (reference)
**Positive**	89 (29.2%)	216 (70.8%)	0.58	0.90 (0.62–1.30)	191 (62.6%)	114 (37.4%)	0.66	0.93 (0.66–1.30)
**HER-2**								
**Negative**	115 (29.6%)	274 (70.4%)		1.00 (reference)	246 (63.2%)	143 (36.8%)		1.00 (reference)
**Positive**	43 (25.1%)	128 (74.9%)	0.29	1.25 (0.83–1.88)	100 (58.5%)	71 (41.5%)	0.29	1.22 (0.85–1.76)
**Ki67**								
**< 14%**	57 (29.2%)	138 (70.8%)		1.00 (reference)	119 (61.0%)	76 (39.0%)		1.00 (reference)
**≥ 14%**	101 (27.7%)	264 (72.3%)	0.70	1.08 (0.74–1.59)	227 (62.2%)	138 (37.8%)	0.79	0.95 (0.67–1.36)

* Two-sided χ^2^ test for the distributions of genotype frequencies.

LN: Axillary lymph node; ER: Estrogen receptor; PR: Progesterone receptor; HER-2: human epidermal growth factor receptor 2.

### Stratified analysis of *DCC* gene variants and BC risk

The results of a stratified analysis are shown in Table 4. When the participants were stratified according to age, differences in *DCC* rs7504990 polymorphism distribution were not statistically significant. However, for *DCC* rs2229080, the GA + AA genotype was significantly expressed lower frequency among older participants (age ≥ 49 years) (OR = 0.65, 95% CI = 0.46–0.91, *P* = 0.01).

**Table 4:** Stratified analyses on association between DCC polymorphisms and breast cancer risk

rs2229080					rs4704853				
Genotypes	Case (*N* = 560)	Control (N = 583)	P*	OR (95%CI)^†^	Genotypes	Case (*N* = 560)	Control (*N* = 583)	P*	OR (95% CI)^†^
	N (%)	N (%)				N (%)	N (%)		
Age < 49					Age < 49				
G/G	73 (27.7%)	94 (34.2%)		1.00 (reference)	C/C	149 (56.7%)	107 (48.6%)		1.00 (reference)
C/G + CC	191 (72.3%)	181 (65.8%)	0.10	0.74 (0.51–1.06)	C/T + T/T	114 (43.3%)	113 (51.4%)	0.08	1.38 (0.96–1.98)
Age ≥ 49					Age ≥ 49				
G/G	85 (28.7%)	118 (38.3%)		1.00 (reference)	C/C	197 (66.3%)	201 (66.3%)		1.00 (reference)
C/G + CC	211 (71.3%)	190 (61.7%)	0.01	0.65 (0.46–0.91)	C/T + T/T	100 (33.7%)	102 (33.7%)	1.00	1.00 (0.71–1.40)

* Two-sided χ^2^ test for the distributions of genotype frequencies.

† Adjusted for age and age at menarche.

### Associations between *DCC* haplotypes and BC risk

We then evaluated the relationship between haplotypes and BC risk. Compared with the common haplotype G_rs2229080_C_rs7504990_A_rs4078288_, the haplotype C_rs2229080_C_rs7504990_A_rs4078288_ was associated with a decreased BC risk (OR = 0.74, 95% CI = 0.61–0.90, *P* = 0.003, Table 5). We also observed a significant association between other haplotypes and BC risk (*P* < 0.001).

**Table 5:** Association between *DCC* haplotypes and breast cancer risk

Haplotypes			Controls (*N* = 1166) *n*, %	Cases (*N* = 1120) *n*, %	OR (95% CI)	*P*
rs2229080	rs7504990	rs4078288				
G	C	A	416 (35.68%)	348 (31.07%)	1.00 (reference)	
G	T	G	296 (25.39%)	226 (20.18%)	1.10(0.88–1.37)	0.424
C	C	A	420 (36.02%)	473 (42.23%)	0.74(0.61–0.90)	0.003
C	T	G	21 (1.80%)	16 (1.43%)	1.10(0.56–2.14)	0.783
Others			13 (1.11%)	57 (5.09%)	0.19(0.10–0.35)	< 0.001

## DISCUSSION

*DCC* is a single-pass transmembrane protein that belongs to the immunoglobulin superfamily, and it is a candidate tumor suppressor gene located on chromosome 18q21 [14]. *DCC* extends more than 1.2 Mb with 29 exons [15], and is the largest tumor suppressor gene identified to date. It is expressed in most normal tissues, including the colonic mucosa, and its expression was greatly reduced or absent in most tested colorectal carcinomas [16]. *DCC* directs cell invasion through the basement membrane, an essential step in the pathological progression of human cancer [17]. Currently, *DCC* is known to be involved in the following biological processes: guidance of developing axons,4 induction of apoptosis [18], control of colorectal tumorigenesis [19], and angiogenesis [20]. *DCC* alterations are apparent in early-stage gastric cancers, emphasizing the importance of this growth regulatory pathway in gastric carcinogenesis [21]. Significant differences were observed between cases without metastasis or local recurrences versus those with metastasis or local recurrences, suggesting that a decrease in *DCC* expression might influence the prognosis of patients with breast cancer [22]. However, no significant role for *DCC* alterations has been found in the pathogenesis of meningiomas [23].

In previous studies, *DCC* gene variants were found to be correlated with some types of cancer. Rai et al. [13] found that the *DCC* haplotype G (rs2229080)-A (rs4078288)-C (rs7504990)-A (rs714) conferred a high risk of gallbladder cancer. The rs714 (A > G) polymorphism contributes to the risk of esophageal and gastric cancers in a Kashmiri population [24]. To date, the possible effects of *DCC* variants had not been studied in BC. In our study, we have provided the initial observation that the *DCC* genotype variants rs2229080 and rs7504990, but not rs4078288, contributed differentially to BC susceptibility. The haplotype C_rs2229080_C_rs7504990_Ars_4078288_ had a significant relationship with decreased BC risk.

A previous study found increased *DCC* expression but a lower rate of axillary lymph node metastasis in mucinous carcinoma than in non-mucinous carcinoma in the human female breast [25]. DCC-negative, HER-2 overexpressing tumors were found to have a marginal influence on the survival duration of patients with BC, indicating that reduced DCC expression and HER-2 overexpression might influence the prognosis of BC [26]. However, that finding differs from our results. According to our study, the *DCC* rs2229080 polymorphism was associated with the lymph node metastasis status. Furthermore, rs2229080 conferred a decreased BC risk in older individuals (≥ 49 years). Our results demonstrate that the rs2229080 polymorphism might play a critical role in BC susceptibility. Moreover, *DCC* rs2229080 polymorphism was reported to occur more frequently in stages C and D in colorectal cancer, and may serve as a prognostic factor for colorectal cancer patients [27]. Rs2229080 polymorphism is located in the coding region, which may decrease DCC gene expression. The expression of DCC is mostly lost or reduced in later clinical stage, higher pathological grade, and poor prognosis in ovarian cancer [28].

Our study had some limitations. First, the sample size was inadequate for a stratified analysis and for an analysis of these associations in patients with mix-type BC. As this is the first report of genetic BC susceptibility related to *DCC* polymorphism, similar studies with larger sample sizes will be needed for further verification. Second, we did not investigate whether predisposing factors, including high-dose radiation exposure, alcohol consumption, and postmenopausal obesity, were associated with the risk of BC because of a lack of such data from both BC patients and healthy controls. The effect of these factors on BC risk should be assessed in a future study.

In conclusion, our case-control study indicates that the rs2229080 and rs7504990 polymorphisms in *DCC* might affect BC susceptibility and progression in Chinese women. Further functional studies and large population-based prospective studies will be needed to provide accurate evidence about the influence of *DCC* variants on BC.

## MATERIALS AND METHODS

### Ethics statement

The study was approved by the Institutional Review Board of Xi'an Jiaotong University (Xi'an, China). Written informed consent was obtained from all participants involved in the study at the time of recruitment.

### Study population

We conducted a hospital-based case-control study of 560 BC patients and 583 cancer-free controls. All participants were recruited without the restrictions of age. All patients met the present pathological criteria of sporadic BC and were diagnosed between January 2013 and October 2014 at the Second Affiliated Hospital of Xi'an Jiaotong University, China. Patients who had other types of cancer were excluded from the study. The controls were randomly selected from among healthy volunteers who underwent annual physical examinations in other hospital departments and who had no prior history of cancer. Controls were frequency matched to patients according to age (±5 years). The methods were performed in accordance with the approved guidelines. All research participants provided written informed consent and were interviewed to obtain detailed information regarding age, sex, ethnicity, religion, place of residence, education, and other potential confounding factors of interest. After the interview, approximately 2 mL of venous blood sample was collected from each healthy control and breast cancer patient before they received the chemotherapy or radiotherapy.

### Genotyping assay

Whole blood samples were placed into EDTA-coated tubes and preserved at −80°C until further use. Genomic DNA was isolated from the whole blood samples using a standard phenol-chloroform extraction method. DNA concentrations were measured via spectrometry (DU530 UV/VIS spectrophotometer; Beckman Instruments, Fullerton, CA, USA). For our study, we selected candidate SNPs in TIM according to HapMap data from a Chinese population. To achieve a power of at least 50%, only SNPs with a minor allele frequency (MAF) > 0.01 was included. Sequenom MassARRAY Assay Design 3.0 Software (Agena Bioscience, San Diego, CA, USA) was used to design a Multiplexed SNP MassEXTEND assay. Finally, three SNPs (rs2229080, rs7504990, and rs4078288) were selected according to Chinese population data from HapMap. *DCC* SNP genotyping was performed using a Sequenom MassARRAY RS1000, according to the manufacturer's instructions. The corresponding primers used for each SNP in this study are listed in Table 6. Sequenom Typer 3.0 Software (Sequenom Inc., San Diego, CA, USA) was used for data analysis.

**Table 6:** Primers used for this study

SNP_ID	1st-PCRP	2nd-PCRP	UEP_SEQ
**rs2229080**	ACGTTGGATGGCTGAGCA TCGGTAAATTCC	ACGTTGGATGTCTTGCCCTC TGGAGCATTG	TGGAGCATTGCAGATCAGC
**rs7504990**	ACGTTGGATGCCAAATCTG CTATTACTCAC	ACGTTGGATGCCAAGTTATG TTGGACAGAG	tCCACACACTTATTGGCAGAT
**rs4078288**	ACGTTGGATGTAGGGAACA AGAGAGAGTGC	ACGTTGGATGCTTCTATTGGT CTAGAGGTG	GGTAATGAGCTATTGGAACTA

### Statistical analyses

The statistical power of the case-control study was calculated using QUANTO software 1.2.4 (University of Southern California, Los Angeles, CA, USA; http://biostats.usc.edu/Quanto.html). HWE was tested by chi-square test for each SNP before the analysis. The Student *t*-test or the *t* ^2^ test was used to compare differences in the distributions of demographic characteristics, selected variables, and genotype frequencies between the cases and controls. We conducted a case-control study for all of the subjects, and then the patients were stratified by age. Odds ratios (ORs) and 95% confidence intervals (CIs) were used to assessed the degree of association between*DCC* rs2229080, rs7504990, and rs4078288 polymorphisms and BC. Allele and genotype frequencies were calculated and compared between patients and controls using SPSS 18.0 for Windows (PASW Statistics; *SPSS* Inc., Chicago, IL, USA). Haplotype analysis was performed with in each gene under the generalized linear models by using the software PHASE. *P* values < 0.05 were considered to indicate statistical significance, and all statistical tests were two-sided.
